# The Effects of Individualized Arousal on Recognition Memory of Words

**DOI:** 10.3390/bs16091594

**Published:** 2026-09-07

**Authors:** Wenling Zhang, Xi Jia

**Affiliations:** School of Psychology, Nanjing Normal University, Nanjing 210024, China; 252302057@njnu.edu.cn

**Keywords:** emotional memory, arousal, valence, recognition, response bias

## Abstract

Emotional information often modulates recognition memory, but the relative roles of arousal and valence remain debated, partly because many studies rely on normative emotion categories rather than individualized affective experience. This study examined whether post-test individualized arousal and valence ratings were associated with recognition-confidence responses to emotional Chinese words and whether the learning task influenced later recognition. Forty participants studied neutral, positive, and negative words under semantic-judgment and recognition-judgment learning conditions. After a 24 h delay, they completed a six-point old/new confidence test and then rated each final-test word for valence and arousal. Linear mixed-effects models showed that individualized arousal was more consistently associated with stronger old-response confidence than individualized valence, with a nonlinear increase at higher arousal levels. Semantic-judgment learning was followed by higher old-item confidence and hit probability than recognition-judgment learning. Descriptive signal-detection summaries indicated that valence-category effects were more evident in false-alarm rates, response criterion, and *d*′ (sensitivity index) estimates than in the primary old-response confidence model. Because affective ratings were collected after recognition, these findings should be interpreted as associative rather than causal, and they highlight the need to distinguish recognition confidence, response bias, and memory sensitivity in emotional memory research.

## 1. Introduction

Emotional information often modulates recognition memory. Compared with neutral information, emotional words, faces, and scenes are often detected more readily, receive preferential attention, and are remembered more vividly or confidently (Amting et al., 2010; Kensinger, 2009; Mather & Nesmith, 2008; Williams et al., 2022). Such emotional modulation is commonly considered adaptive because it may help individuals prioritize information related to threat, reward, well-being, or future behavior (Öhman & Mineka, 2001). Emotional material can also increase false recognition, strengthen feelings of familiarity, or shift response criteria. Therefore, understanding how emotion is related to later recognition requires distinguishing recognition confidence, response tendency, and memory sensitivity.

A central issue concerns the relative and joint contributions of arousal and valence. Valence refers to how pleasant or unpleasant a stimulus is, whereas arousal refers to the degree of activation or emotional intensity it evokes (MacMillan et al., 2022). Arousal-centered accounts propose that emotionally intense stimuli receive prioritized processing and are more strongly associated with later memory-related responses across both positive and negative domains (Anderson et al., 2003; Anderson, 2005; Mather et al., 2004; Mather & Sutherland, 2009, 2011). In contrast, valence-centered accounts emphasize that positive and negative information may differ in motivational significance, attentional consequences, and retrieval-related outcomes (Bowen et al., 2018; Pereira et al., 2023). These perspectives are not mutually exclusive, but their predictions are difficult to distinguish because valence and arousal often covary in stimulus materials: positive and negative stimuli are typically more arousing than neutral stimuli. Apparent effects of emotional category may, therefore, reflect valence, arousal, variance shared by the two dimensions, or their interaction.

Distinguishing these possibilities requires estimating the unique association of each dimension and testing their interaction without assuming independence or interaction in advance. Behavioral studies provide mixed support for dimension-specific effects: some attribute emotional-memory advantages primarily to arousal (Bradley et al., 1992), whereas others report valence effects after controlling for arousal (Adelman & Estes, 2013), no reliable valence effects (MacMillan et al., 2022), or simultaneous but functionally distinct contributions of both dimensions: arousal shows a relatively monotonic positive association with memory, whereas valence follows a U-shaped pattern with better memory for positive and negative than neutral words (Gao et al., 2024). Arousal-related effects may also depend on stimulus valence; experimentally crossed audiovisual contexts have yielded valence–arousal interactions whose direction and size differed across memorization, discrimination, and fixation outcomes (Du Bruyn et al., 2026; Mickley Steinmetz et al., 2010). At physiological and neural levels, valence and arousal show partly distinct response patterns and neural systems (Lang et al., 1993; Kensinger & Corkin, 2004; Xu et al., 2015; Amini et al., 2026). Thus, neither general independence nor invariant interaction can be assumed; the present study therefore estimated unique associations of individualized valence and arousal with recognition responses and compared additive and interaction models.

The validity of these comparisons also depends on how emotional experience is measured. Many studies select stimuli from standardized databases and classify them as neutral, positive, or negative on the basis of normative ratings provided by independent samples (Jia et al., 2019; Schaefer et al., 2011). Although this approach facilitates stimulus selection and experimental control, it may not fully capture each participant’s experience. The same word may be mildly unpleasant to one participant but highly unpleasant or arousing to another. This limitation is especially relevant to emotional word recognition, in which affective associations may also depend on lexical characteristics, task demands, and individual differences (Ferré et al., 2025; Haro et al., 2024, 2025). Treating valence and arousal as individualized continuous ratings can therefore complement designs based on normative emotional categories and provide a more sensitive assessment of their trial-level associations with recognition responses. Because the individualized ratings in the present study were collected after the recognition test, however, they were treated as post-test subjective appraisals rather than direct measures of affective experience during encoding.

A more sensitive characterization of subjective affect does not resolve the second interpretive issue: whether stronger emotional-recognition responses reflect enhanced memory sensitivity, a shift in response bias, or both. Dougal and Rotello (2007) provided particularly direct evidence for a response-bias account. In experiments using remember–know judgments and confidence ratings, emotional words elicited a greater proportion of subjective “remember” responses. However, receiver operating characteristic (ROC) analyses and model comparisons indicated that this difference arose primarily from a shift in response bias rather than from enhanced memory sensitivity or a greater contribution from a high-threshold recollection process. Their findings demonstrate that more frequent, more confident, or phenomenologically stronger “old” responses to emotional material do not by themselves establish enhanced recollection. Emotional words may instead increase the tendency to endorse both studied and unstudied items as old, thereby increasing hit rates as well as false-alarm rates. Related research has similarly shown that emotional materials, particularly negative or highly arousing materials, can shift response criteria, increase false recognition, or affect response bias alongside correct recognition (Brainerd et al., 2010; Cooper & Shah, 2025; Hellenthal et al., 2019; Howe et al., 2010). Analyses of emotional recognition should therefore consider not only confidence and the frequency of old-oriented responses but also signal-detection indices that distinguish memory sensitivity from response bias. Accordingly, the present study examined old-response confidence together with false-alarm rates, response criterion, and *d*′ to evaluate whether emotional-recognition patterns were more consistent with differences in memory sensitivity, response bias, or both.

Beyond affective experience and response processes, subsequent recognition may also be shaped by the cognitive operations performed during learning. According to the levels-of-processing framework, judging whether a word is abstract or concrete requires semantic analysis and should produce richer, more distinctive memory representations than a brief recognition judgment (Craik & Lockhart, 1972; Craik & Tulving, 1975). Although retrieval-based learning often improves retention, its benefits are generally greater when retrieval requires effortful reconstruction (Karpicke, 2017; Rowland, 2014). A relatively undemanding recognition judgment may instead rely substantially on familiarity and may therefore provide less elaboration than a semantic judgment. Encoding-task effects on emotional memory may also depend on retention interval and task context (Jia et al., 2024), and retrieval practice may be less beneficial when task demands consume available cognitive resources (Zheng et al., 2023). The learning-task manipulation was therefore included alongside the individualized-affect analyses so that the same recognition-confidence outcome could be examined in relation to both an experimentally controlled encoding operation and participant-specific post-test affective appraisals. We predicted a general advantage for semantic-judgment learning over recognition-judgment learning. However, we had no directional basis for predicting that the learning task would strengthen or weaken the associations of individualized arousal or valence with recognition confidence: the semantic task directed attention to concreteness rather than emotional properties, and the individualized affective ratings were collected only after the final recognition test. Learning-task interactions with arousal or valence were therefore not treated as primary directional hypotheses.

The present study integrated these components by examining whether post-test individualized arousal and valence ratings were associated with recognition-confidence responses to emotional Chinese words and whether the experimentally manipulated learning task influenced subsequent recognition. Forty participants studied neutral, positive, and negative words under semantic-judgment and recognition-judgment learning conditions. After a 24 h delay, they completed a six-point old/new confidence test and then rated every final-test word for valence and arousal. Trial-level linear mixed-effects models estimated the associations of individualized affective ratings with old-response confidence while accounting for participant- and item-level variability and learning condition. Additive and valence–arousal interaction models were compared, and possible nonlinearity in the arousal–confidence association was also examined. Descriptive signal-detection summaries were used to determine whether valence-category differences were evident in false-alarm rates, response criterion, *d*′, or some combination of these measures. Because the affective ratings were collected after recognition, their associations with recognition responses were interpreted as involving post-test subjective appraisals rather than causal effects of affective experience during encoding. Based on prior evidence, we predicted that higher individualized arousal would be associated with stronger old-response confidence and that semantic-judgment learning would produce stronger subsequent recognition responses than recognition-judgment learning.

## 2. Materials and Methods

### 2.1. Participants

Forty right-handed participants (19 females; *M*age = 23.12 years, *SD* = 2.05) from Hangzhou Normal University participated in the experiment. All had normal or corrected-to-normal vision. Each participant signed an informed consent form and received monetary compensation for their participation. This study was approved by the Ethics Committee of Hangzhou Normal University.

### 2.2. Stimuli

Five hundred and four words were selected from the Chinese Affective Words System (CAWS; Wang et al., 2008), including 168 neutral words (60 nouns, 54 verbs, 54 adjectives), 168 positive words (60 nouns, 54 verbs, 54 adjectives), and 168 negative words (60 nouns, 54 verbs, 54 adjectives). The rating scores of valence, arousal, and familiarity for all words were obtained from the CAWS database. Valence ratings ranged from 1 (extremely unpleasant) to 9 (extremely pleasant), arousal ratings ranged from 1 (not at all arousing) to 9 (extremely arousing), and familiarity ratings ranged from 1 (extremely unfamiliar) to 9 (extremely familiar). Normative arousal ratings were higher for positive words (*M* = 5.17, *SD* = 0.54) and negative words (*M* = 5.24, *SD* = 0.61) than for neutral words (*M* = 4.25, *SD* = 0.58), *p*s < 0.001, with no significant difference between positive and negative words, *p* > 0.05. Normative valence ratings were highest for positive words (*M* = 6.56, *SD* = 0.32), intermediate for neutral words (*M* = 5.29, *SD* = 0.50), and lowest for negative words (*M* = 3.25, *SD* = 0.45), *p*s < 0.001. Normative familiarity ratings differed modestly across categories (neutral: *M* = 5.26, *SD* = 0.46; positive: *M* = 5.30, *SD* = 0.51; negative: *M* = 5.12, *SD* = 0.48), *F*(2, 501) = 6.28, *p* = 0.002, *η*^2^ = 0.024. Thus, familiarity matching should be regarded as approximate rather than exact. Words were presented in white on a black background of a 17-inch monitor (1024 × 768 pixels), covering approximately 2.5 degrees horizontally by 2.5 degrees vertically.

### 2.3. Procedure

Participants were seated approximately 80 cm from the monitor in an acoustically attenuated room. The experiment consisted of six word-learning blocks, including three semantic-judgment learning blocks and three recognition-judgment learning blocks. The order of the six blocks was counterbalanced across participants so that the two learning conditions were distributed throughout the session. Each block consisted of an initial study phase followed by either a semantic-judgment learning task or a recognition-judgment learning task. Across the six initial study phases, 252 words were presented and later served as old words in the final recognition test.

**Figure 1:**
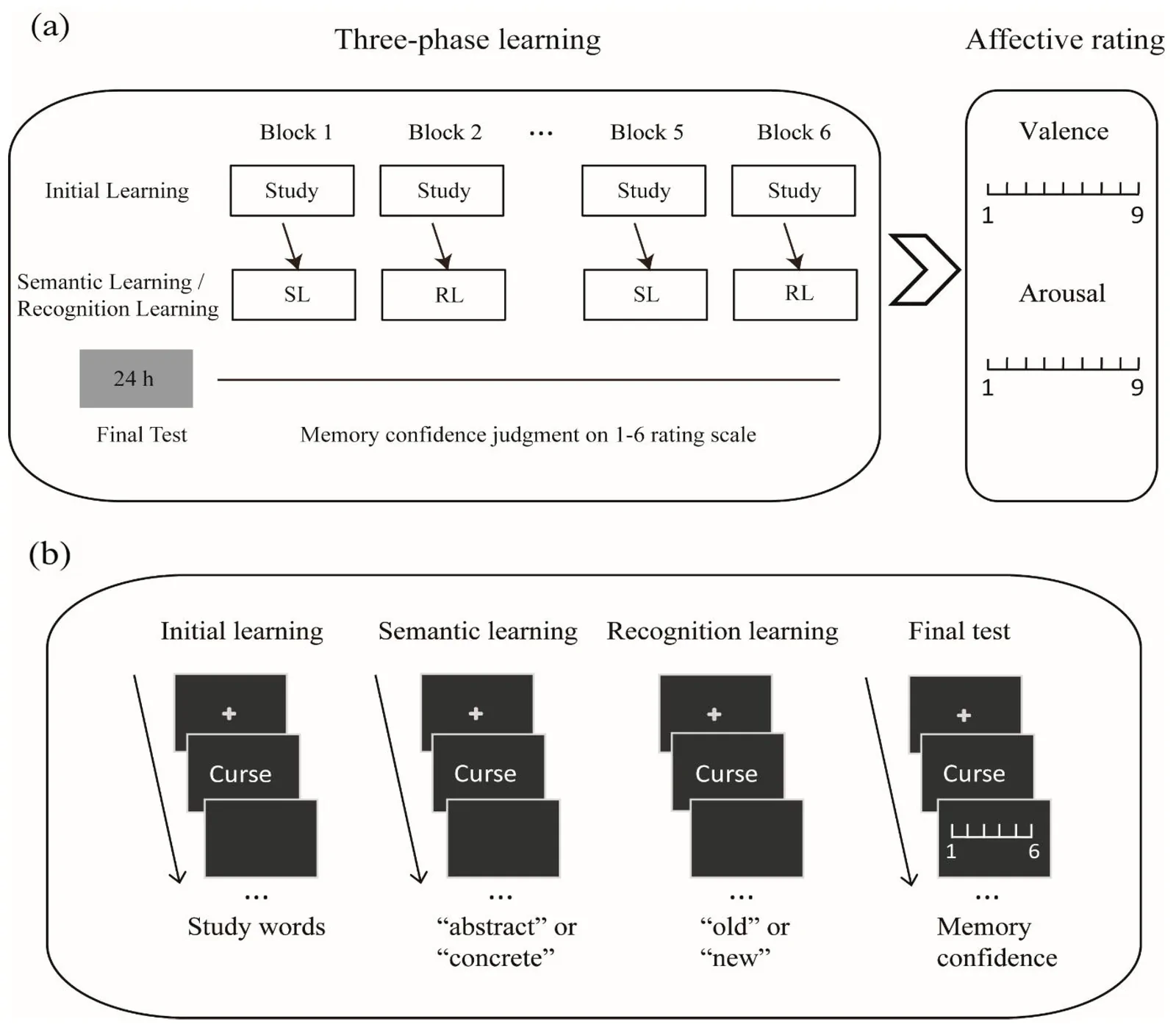
(**a**) Participants completed six word-learning blocks. Each block consisted of an initial study phase followed by either a semantic-judgment learning task or a recognition-judgment learning task. Words presented during the initial study phases later served as old items in the final recognition test. After a 24 h delay, participants completed a six-point old/new confidence test including old items and completely new items. They then rated all final-test words for valence and arousal. (**b**) Trial structure for the main task phases. Participants studied words during the initial study phase, made abstract/concrete judgments during the semantic-judgment learning task, made old/new judgments during the recognition-judgment learning task, and finally made six-point memory-confidence judgments during the delayed recognition test. Source: Created by the authors.

The remaining 252 words were divided into two sets. One set of 126 words was used only as new items in the intermediate semantic-judgment or recognition-judgment learning tasks and did not appear in the final recognition test. The other set of 126 words was reserved as completely new items for the final recognition test and had not appeared in any previous phase of the experiment. Thus, the final recognition test included 252 old words from the initial study phases and 126 completely new words. The final-test items were balanced across emotional categories, with equal numbers of neutral, positive, and negative words (Figure 1).

In each semantic-judgment learning block, the initial study phase included 42 words, consisting of 14 neutral, 14 positive, and 14 negative words. Each trial began with a fixation cross presented at the center of the screen for a jittered interval of 1000–1300 ms, followed by a word presented for 500 ms. Participants were instructed to study each word. The subsequent semantic-judgment learning task included 63 words: the 42 words presented in the immediately preceding initial study phase and 21 new words, consisting of 7 neutral, 7 positive, and 7 negative words. In each trial, a word was presented for 500 ms, followed by a fixation cross for a jittered interval of 1000–1300 ms. Participants made an abstract/concrete judgment about the meaning of each word. Thus, this task required semantic analysis rather than an explicit old/new memory judgment. In each recognition-judgment learning block, the initial study phase was identical to that in the semantic-judgment learning block, except that different stimuli were used. The subsequent recognition-judgment learning task included 63 words: the 42 words presented in the immediately preceding initial study phase and 21 new words, consisting of 7 neutral, 7 positive, and 7 negative words. In each trial, a word was presented for 500 ms, followed by a fixation cross for a jittered interval of 1000–1300 ms. Participants made an old/new judgment for each word. They were instructed to press the F key for an old response if the word had appeared in the immediately preceding initial study phase and the J key for a new response if the word had not appeared in that phase.

Twenty-four hours after completing the six learning blocks, participants completed the final recognition test using a six-point old/new confidence scale. The 252 old words included 126 words from the semantic-judgment learning condition and 126 words from the recognition-judgment learning condition, with each learning condition containing 42 neutral, 42 positive, and 42 negative old words. The 126 completely new words included 42 neutral, 42 positive, and 42 negative words. For analytic balancing, these final-test new words were divided into semantic-judgment and recognition-judgment sets matched by emotional category, so that each learning-condition label contained an equal number of old and new test items from each emotional category. This assignment was used only for analysis and did not indicate that the new words had been presented during learning.

In each final-test trial, a word was presented for 1000 ms, followed by a fixation cross for a jittered interval of 1000–1300 ms. A response screen was then presented, and participants made a memory-confidence judgment using the six-point scale. Ratings from 1 to 3 indicated new judgments with decreasing confidence in the new response (1 = 100%, 2 = 75%, and 3 = 50% confident new), whereas ratings from 4 to 6 indicated old judgments with increasing confidence in the old response (4 = 50%, 5 = 75%, and 6 = 100% confident old). Thus, ratings 3 and 4 were adjacent low-confidence responses on opposite sides of the old/new decision boundary, rather than equivalent responses.

After the final recognition test, participants rated all final-test words for valence and arousal using nine-point scales. The valence scale ranged from 1 (extremely unpleasant) to 9 (extremely pleasant), and the arousal scale ranged from 1 (not arousing at all) to 9 (extremely arousing). The rated words included the 252 old words and the 126 completely new words from the final recognition test. Collecting affective ratings after the memory test ensured that ratings were obtained for the exact final-test items, but it also means that recognition decisions, processing fluency, and confidence may have influenced subsequent affective ratings. Therefore, individualized valence and arousal were treated as post-test subjective ratings associated with recognition judgments, rather than as experimentally manipulated encoding variables.

For all judgment phases, the response display remained visible until the participant responded. Therefore, response time was variable rather than fixed. The fixed durations reported above refer only to the word-presentation and fixation intervals. This procedure applied to the semantic-judgment task, the recognition-judgment task, the final recognition-confidence test, and the post-test affective-rating task.

### 2.4. Data Analyses

#### 2.4.1. Descriptive and Signal-Detection Analyses

The planned final recognition test contained 15,120 trials, corresponding to 40 participants × 378 final-test words. Trials with missing or invalid final-test responses were excluded before analysis. The final analysis dataset contained 14,897 valid trials, including 9943 old-item trials and 4954 new-item trials. Descriptive checks first summarized the completeness of the data, the distribution of old and new trials, and whether post-test individualized affective ratings preserved the intended emotional category structure. Individualized valence and arousal ratings were summarized at the participant level and compared across the three normative valence categories using repeated-measures analyses. To evaluate whether the individualized ratings were consistent with the original CAWS stimulus norms, item-level mean individualized ratings were correlated with CAWS normative valence and arousal ratings across the 378 final-test words. Because individualized affective ratings were collected after the recognition test, they were treated as post-test subjective predictors associated with recognition judgments rather than as experimentally manipulated encoding variables.

Traditional recognition summaries were then computed before fitting trial-level models. For old items, old-response confidence was defined as the six-point response score, with higher scores indicating stronger endorsement that the word had been studied. Hit rate was defined as the proportion of old items receiving responses from 4 to 6. For new items, false-alarm rate was defined as the proportion of new items receiving responses from 4 to 6. Old-item confidence and hit rates were analyzed using participant-level repeated-measures analyses with Learning Condition (semantic judgment, recognition judgment) and Valence Category (negative, neutral, positive) as within-participant factors. False-alarm rates were analyzed across valence categories. Learning-condition labels for new items were analytic pseudo-labels used only to balance item assignment across cells and did not indicate the actual learning histories for new words.

Signal-detection indices were computed to help distinguish memory sensitivity from response bias at the categorical level. For each participant, learning-condition label, and valence category, *d*′ and criterion *c* were computed from hit and false-alarm proportions after applying the loglinear correction: (count + 0.5)/(N + 1). Higher *d*′ values indicate better discrimination between old and new items, whereas more negative criterion *c* values indicate a more liberal tendency to respond “old”. These signal-detection summaries were used as descriptive checks of sensitivity and response criterion, rather than as a full continuous-arousal decomposition of latent sensitivity and bias.

#### 2.4.2. Linear Mixed-Effects Models

Trial-level linear mixed-effects models were fitted in R using the lme4 package (Bates et al., 2015a). The dependent variable was the six-point old/new confidence response, treated as an approximately continuous old-response score. Because the scale combines response direction with confidence strength, higher values indicate stronger old-oriented responding rather than uniformly better memory accuracy: higher scores reflect more confident “old” responses, which are correct for old items and constitute false alarms for new items. Individualized valence and arousal ratings were standardized separately within each participant by subtracting the participant-specific mean and dividing by the participant-specific standard deviation (Schielzeth, 2010). Quadratic terms were formed from these standardized scores. Consequently, coefficients for valence and arousal represent within-participant associations: they estimate how old-oriented response strength changed when an item was rated above or below that participant’s own average. Learning condition was coded with recognition judgment as the reference level, and item status was coded with old items as the reference level. All interaction models retained the corresponding lower-order terms.

Five candidate fixed-effect structures were evaluated. Model 1 included linear and quadratic arousal terms, learning condition, and item status. Model 2 added linear valence to Model 1. Model 3 added the conventional linear Valence × Arousal interaction to Model 2. Model 4 was a second-order response-surface model containing linear and quadratic terms for both valence and arousal, together with the Valence × Arousal interaction. Model 5 was an exploratory extension of Model 3 that added the Valence × Arousal^2^ term. All affective terms were based on within-participant standardized ratings, and all models included crossed random intercepts for participant and stimulus item. Models that differed in fixed-effect structure were first fitted using maximum likelihood. The relevant nested models were compared using likelihood-ratio tests, while the Akaike information criterion (AIC) and Bayesian information criterion (BIC) were used as complementary indices of relative fit and parsimony. The model retained for reporting was then refitted using restricted maximum likelihood (REML) for coefficient estimation. For fixed effects represented by a single coefficient, Wald *χ*^2^ statistics were computed as (*β*/*SE*)^2^, corresponding to one-degree-of-freedom tests, and 95% Wald confidence intervals were reported. The full candidate-model formulas, maximum-likelihood model comparisons, and restricted maximum-likelihood fixed-effect estimates are reported in Supplementary Analysis S1.

Because the standardized arousal predictor was centered at zero, its linear coefficient in models containing Arousal^2^ represents the local arousal slope at the participant-specific mean (z = 0). The quadratic term was included as an exploratory test of curvature rather than as a prespecified directional prediction. Theoretical accounts allow arousal–cognition relations to vary across the arousal range (Yerkes & Dodson, 1908; Aston-Jones & Cohen, 2005) and suggest that arousal does not affect all representations uniformly (Mather & Sutherland, 2011), but they did not determine the sign or turning point of the function tested here. A positive quadratic coefficient therefore indicates upward curvature, not necessarily a monotonic increase across the entire range. Because the nonlinear analysis was not preregistered, its estimated form was treated as exploratory and as requiring confirmation.

The directional prediction for the learning condition applied only to studied items: semantic-judgment learning was expected to yield stronger subsequent old-item responses than recognition-judgment learning. This prediction was evaluated primarily in the participant-level analyses of old-item response scores and hit rates. Learning condition was nevertheless retained in the all-trial mixed-effects models as a design-control factor. Because new items had no learning history and their condition labels were assigned only for analytic balancing, the all-trial learning-condition coefficient was not interpreted as an encoding effect on new items or as a stand-alone estimate of the old-item learning effect. No directional Learning Condition × Arousal or Learning Condition × Valence hypothesis was specified, and these interactions were not included in the candidate mixed-effects models. The Learning Condition × Valence Category effect in the participant-level analyses was evaluated without a directional prediction.

Crossed random intercepts accounted for repeated observations within participants and for repeated testing of the same words across participants. Because the dataset contained 40 participant clusters and the candidate models already included several correlated polynomial terms, the analyzed models were restricted to random intercepts rather than a maximal random-slope structure (Bates et al., 2015b). This specification does not model participant- or item-specific variation in the affective slopes; the fixed effects were therefore interpreted as average associations under an intercept-only random-effects structure and considered together with the participant-level analyses.

#### 2.4.3. Exploratory Leave-One-Participant-Out Cross-Validation

Leave-one-participant-out cross-validation was conducted as an exploratory assessment of fixed-effect generalization. The mixed-effects analysis estimates associations and their uncertainty in the observed dataset while accounting for repeated observations within participants and items. Cross-validation addresses a related but distinct question: whether the population-level fixed effects estimated from other participants retain any ability to predict the responses of a participant who did not contribute to model fitting. Thus, the analysis was intended to complement the inferential mixed-effects results with an out-of-sample participant check, not to replace the mixed-effects analysis or serve as a central machine-learning contribution.

The procedure can be summarized in four steps and was applied separately to each model being evaluated. First, one participant was excluded. Second, the model under evaluation was fitted to the trial-level data from the remaining 39 participants. Third, the fixed-effect coefficients from this training set were used to generate predicted responses for the excluded participant. These held-out predictions were generated from the fixed effects only, with random-effect contributions set to zero, because the aim was to evaluate whether the population-level fixed-effect structure generalized to an unseen participant. Fourth, predictive performance for that participant was summarized as the Pearson correlation between observed and fixed-effect-predicted response scores. These steps were repeated until each of the 40 participants had served once as the held-out participant, and the participant-level correlations were averaged. A permutation test evaluated whether the observed mean correlation exceeded chance by randomly disrupting the correspondence between held-out responses and predictions. The full model was also compared with a no-arousal model and a status-only model under the same folds to assess whether the arousal terms added held-out predictive information beyond the basic task structure.

## 3. Results

### 3.1. Descriptive and Signal-Detection Results

**Figure 2:**
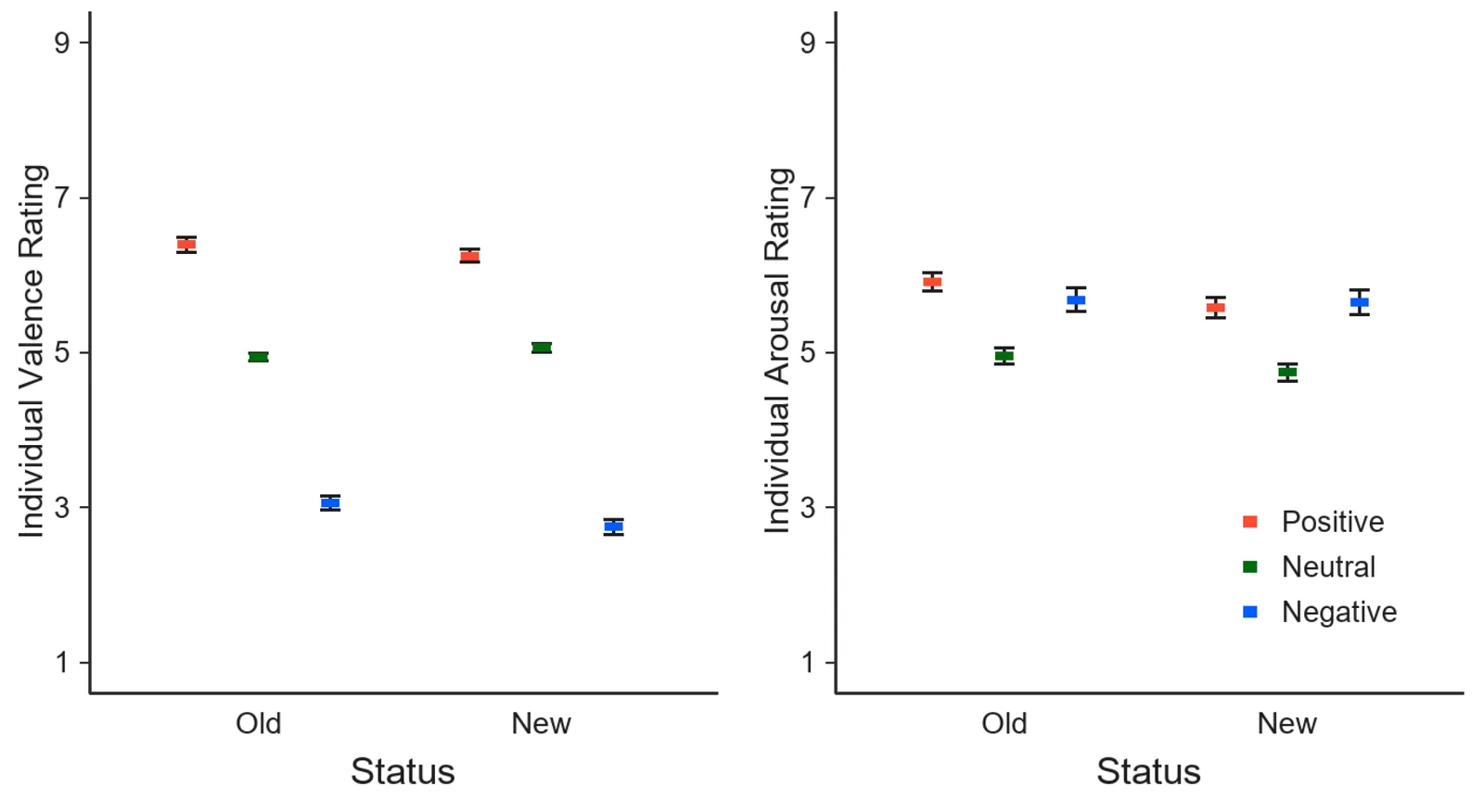
Post-test individualized valence and arousal ratings for positive, neutral, and negative words by old/new item status. Points represent participant-level means averaged across participants, and error bars represent standard errors across participants. Source: Generated by the authors from the present data.

The final analysis dataset included 14,897 valid trials out of 15,120 planned final-test trials, corresponding to a valid-trial rate of 98.53%. The retained dataset included 9943 old-item trials and 4954 new-item trials. Post-test individualized affective ratings broadly preserved the intended emotional category structure. Individualized valence ratings differed strongly across valence categories, *F*(2, 78) = 419.51, *p* < 0.001. Negative words received the lowest valence ratings (*M* = 2.95, *SD* = 0.57), neutral words received intermediate ratings (*M* = 4.98, *SD* = 0.31), and positive words received the highest ratings (*M* = 6.34, *SD* = 0.57). Individualized arousal ratings also differed across valence categories, *F*(2, 78) = 23.52, *p* < 0.001. Negative words (*M* = 5.67, *SD* = 0.98) and positive words (*M* = 5.79, *SD* = 0.78) were rated as more arousing than neutral words (*M* = 4.88, *SD* = 0.65). After matching final-test items to the CAWS source list, item-level mean individualized ratings were strongly correlated with CAWS normative valence ratings, *r* = 0.91, and moderately correlated with CAWS normative arousal ratings, *r* = 0.58. The same category-level rating pattern was visually apparent for both old and new final-test items (Figure 2). Thus, although individualized affective ratings were collected after recognition, they were broadly consistent with the normative stimulus categories while still preserving participant-specific variation for the mixed-effects analyses.

For old items, old-response confidence was higher after semantic-judgment learning (*M* = 4.67, *SD* = 0.60) than after recognition-judgment learning (*M* = 4.52, *SD* = 0.66), *F*(1, 39) = 8.19, *p* = 0.007, *η*_p_^2^ = 0.174. Valence category also affected old-item old-response confidence, *F*(2, 78) = 3.89, *p* = 0.024, *η*_p_^2^ = 0.091, whereas the Learning Condition × Valence Category interaction was not significant, *F*(2, 78) = 0.07, *p* = 0.932. A parallel hit-rate analysis showed that old items from the semantic-judgment condition were more likely to receive old responses (*M* = 0.767, *SD* = 0.130) than old items from the recognition-judgment condition (*M* = 0.735, *SD* = 0.145), *F*(1, 39) = 8.31, *p* = 0.006, *η*_p_^2^ = 0.176. The main effect of valence category was marginal, *F*(2, 78) = 2.77, *p* = 0.069, and the interaction was not significant, *F*(2, 78) = 0.33, *p* = 0.717. Semantic-judgment learning was generally associated with numerically higher old-response confidence and hit rates than recognition-judgment learning across valence categories (Table 1). False-alarm rates showed a clear valence-category effect. New negative words produced the highest false-alarm rate (*M* = 0.545, *SD* = 0.189), followed by positive words (*M* = 0.504, *SD* = 0.192) and neutral words (*M* = 0.453, *SD* = 0.164), *F*(2, 78) = 12.77, *p* < 0.001, *η*_p_^2^ = 0.247. Signal-detection summaries further showed a significant valence effect on *d*′, *F*(2, 78) = 5.92, *p* = 0.004, *η*_p_^2^ = 0.132. Descriptively, *d*′ was highest for neutral words (*M* = 0.83, *SD* = 0.44), intermediate for positive words (*M* = 0.75, *SD* = 0.49), and lowest for negative words (*M* = 0.62, *SD* = 0.45). Criterion c also differed across valence categories, *F*(2, 78) = 10.12, *p* < 0.001, *η*_p_^2^ = 0.206. Criterion values were most liberal for negative words (*M* = −0.44, *SD* = 0.45), followed by positive words (*M* = −0.40, *SD* = 0.42), and were least liberal for neutral words (*M* = −0.29, *SD* = 0.42).

Together, the descriptive and signal-detection analyses indicate that emotional valence was associated with stronger old-response tendencies, higher false-alarm rates, and more liberal response criteria, rather than a simple enhancement of memory sensitivity.

### 3.2. Linear Mixed-Effects Model Results

Trial-level mixed-effects models evaluated whether participant-relative valence and arousal ratings explained old-oriented response strength beyond learning condition and item status. The candidate fixed-effect structures were compared under maximum likelihood, and the retained model was refitted under restricted maximum likelihood. The complete candidate-model definitions, maximum-likelihood fit indices and nested model comparisons, and restricted maximum likelihood (REML) fixed-effect estimates for the five candidate linear mixed-effects models are reported in Supplementary Analysis S1.

Model comparisons supported the arousal-based structure as the primary reporting model. Adding individualized valence to Model 1 did not improve fit, likelihood-ratio *χ*^2^(1) = 0.81, *p* = 0.369, ΔAIC = +1.19, and ΔBIC = +8.80. Adding the linear Valence × Arousal interaction to Model 2 likewise did not improve fit, likelihood-ratio *χ*^2^(1) = 0.22, *p* = 0.636, ΔAIC = +1.78, and ΔBIC = +9.39. Relative to Model 1, the response-surface model added linear valence, quadratic valence, and the Valence × Arousal term but did not improve fit, likelihood-ratio *χ*^2^(3) = 1.44, *p* = 0.695, ΔAIC = +4.56, and ΔBIC = +27.38. Model 5, which added Valence × Arousal^2^ to Model 3, improved the likelihood-ratio comparison, *χ*^2^(1) = 6.74, *p* = 0.009, and reduced AIC by 4.74 points, but increased BIC by 2.87 points. Because this improvement involved an exploratory higher-order interaction and the information criteria disagreed, Model 5 was not adopted as the primary reporting model; the more parsimonious Model 1 was retained.

The retained model included the within-participant standardized linear and quadratic arousal terms, learning condition, and item status as fixed effects, with crossed random intercepts for participants and stimulus items:

Response ~ Arousal_z + Arousal_z^2^ + Learning Condition + Item Status + (1 | Participant) + (1 | Item)

**Figure 3:**
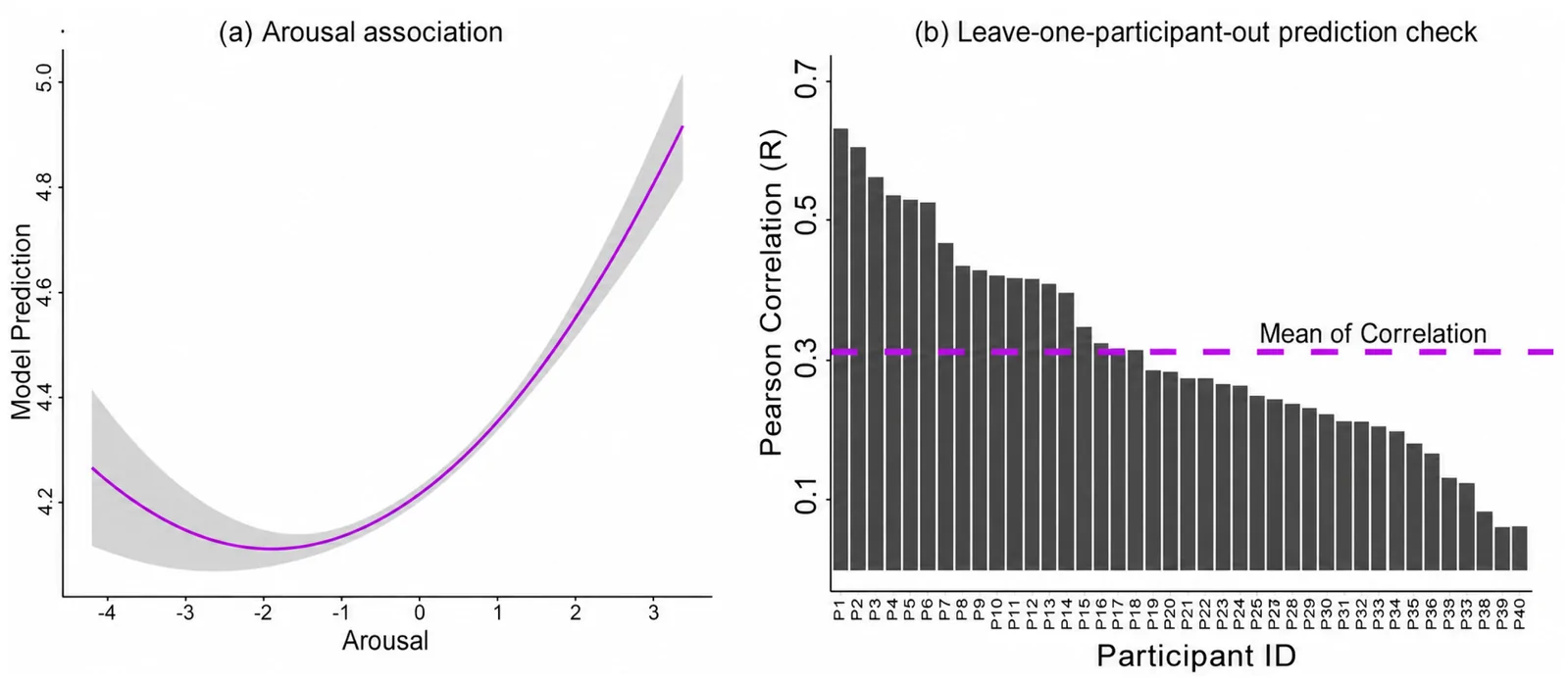
Model-based arousal association and fixed-effects leave-one-participant-out prediction check. (**a**) Predicted old-response confidence from the final linear mixed-effects model as a function of within-participant standardized individualized arousal. The purple curve represents the model-predicted values, and the gray shaded area indicates the confidence interval. The arousal function was quadratic: old-response confidence changed relatively little at the low-arousal end, whereas highly arousing items tended to receive stronger old-oriented responses than low- or moderate-arousal items. Error bands and bars summarize descriptive standard errors across participant-level predicted values rather than from a separate latent sensitivity estimate. (**b**) Participant-level leave-one-participant-out fixed-effect prediction performance for the full model. Gray bars represent individual participants’ Pearson correlation coefficients between predicted and observed responses, and the purple dashed horizontal line indicates the mean correlation, r(predicted, observed) = 0.312. Source: Generated by the authors from the present mixed-effects and cross-validation analyses.

When refitted using restricted maximum likelihood, the linear arousal coefficient at z = 0 was positive, Wald *χ*^2^(1) = 17.95, *p* < 0.001, 95% CI [0.032, 0.088]. The quadratic coefficient was also positive, Wald *χ*^2^(1) = 7.46, *p* = 0.006, 95% CI [0.008, 0.047]. Together, these coefficients indicate upward curvature rather than a constant positive slope across the entire arousal range. The fitted curve had a shallow minimum in the lower portion of the observed range and then increased progressively more steeply at higher arousal levels (Figure 3a). Thus, within participants, items rated as highly arousing relative to the participant’s own average tended to receive the strongest old-oriented responses; the fitted relation at low-to-moderate arousal was not strictly monotonic. Because arousal was rated after the recognition response and the outcome combines response direction with confidence, this pattern does not establish that arousal caused better memory accuracy.

The learning-condition label coefficient was positive, Wald *χ*^2^(1) = 23.84, *p* < 0.001, 95% CI [0.072, 0.169]. With recognition judgment as the reference level, trials labeled as semantic judgment had higher old-response scores. However, this contrast represented a genuine learning manipulation only for old items; the condition labels assigned to new items were analytic pseudo-labels. Accordingly, the substantive evidence for a semantic-judgment advantage came primarily from the participant-level old-item analyses, which showed higher old-item confidence and hit rates after semantic-judgment than recognition-judgment learning. The all-trial coefficient was treated as a design-control term rather than as evidence of an encoding effect on new items. Item status showed the largest effect, Wald *χ*^2^(1) = 289.38, *p* < 0.001, 95% CI [−1.195, −0.948]. With old items as the reference level, new items received substantially lower old-response scores.

Linear individualized valence did not provide sufficient incremental explanatory value after participant-relative arousal, learning condition, and item status were taken into account, and the conventional linear Valence × Arousal interaction was also unsupported. These results should not be interpreted as evidence that valence was behaviorally irrelevant. In the participant-level and signal-detection analyses, normative valence-category differences were more evident in false-alarm rates, criterion c, and *d*′ than in the trial-level model of old-response strength. The exploratory Valence × Arousal^2^ result was retained only as a supplementary finding. Taken together, the primary model identified participant-relative arousal as the more consistent trial-level correlate of old-oriented response strength, whereas valence-category effects were more apparent in categorical measures of response bias and memory sensitivity. The retained-model estimates are summarized below (Table 2).

### 3.3. Exploratory Leave-One-Participant-Out Cross-Validation Results

Finally, leave-one-participant-out cross-validation was used as a supplementary held-out participant prediction check rather than as a central machine-learning contribution. The full model produced an average correlation between observed and fixed-effect-predicted responses of *r*(predicted, observed) = 0.312, and the permutation test indicated that this value was above chance (*p* = 0.001). However, the no-arousal model (mean *r* = 0.308) and the status-only model (mean *r* = 0.306) performed very similarly. The full model exceeded the no-arousal model by only Δ*r* = 0.004 and the status-only model by only Δ*r* = 0.006. Thus, the leave-one-participant-out results indicate that the overall fixed-effect structure captured reliable response regularities, but most predictable variance came from the basic task structure, especially item status, and the incremental predictive contribution of individualized arousal was small (Figure 3b).

The above-chance correlation indicates that the population-level fixed-effect structure captured some ordering of trial responses that generalized across participants. It should not be interpreted as perfect calibration, as accurate participant-specific prediction, or as evidence that every component of the full model contributed equally. In particular, the very small differences between the full and reduced models show that adding individualized arousal produced little incremental predictive gain for a previously unseen participant, even though the overall fixed-effect predictions were reliably above chance.

## 4. Discussion

The present study combined participant-specific affective ratings, an experimentally manipulated learning task, and complementary indices of recognition behavior to clarify how emotional information was expressed in delayed word recognition. The findings do not support a single, unitary emotional-memory advantage. Instead, arousal, valence, and learning condition were associated with different components of recognition performance. Participant-relative post-test arousal was most consistently associated with trial-level old-oriented response strength, normative valence-category differences were more clearly reflected in false-alarm rates, response criterion, and discrimination, and the learning-task manipulation was most interpretable in analyses restricted to studied items. This differentiation is important because stronger endorsement of an item as “old” is not equivalent to greater memory accuracy, and because experimentally manipulated learning history and post-test subjective ratings support different kinds of inference.

The association involving individualized arousal is broadly consistent with accounts proposing that emotionally intense information receives prioritized processing and can influence later memory-related judgments (Anderson et al., 2003; Anderson, 2005; Mather et al., 2004; Mather & Sutherland, 2011). By treating arousal as participant-relative, the present analysis examined whether words rated as more arousing than a participant’s own average elicited stronger old-oriented responses from that participant. This approach captures subjective variability that normative classifications may miss. However, because arousal was rated after recognition, the association may partly reflect recognition confidence, familiarity, or processing fluency. It should therefore be interpreted as an alignment between post-test arousal and old-oriented responding, not as causal evidence that arousal enhanced memory. This caution is important because higher six-point scores indicated confident correct “old” responses for studied items but confident false alarms for new items. Thus, stronger old-oriented responding cannot be equated with greater accuracy. The signal-detection results showed that emotional category differences were also evident in false-alarm rate, criterion c, and *d*′, indicating that emotional category was related to response bias and discrimination. Consistent with Dougal and Rotello’s (2007) response-bias account, emotional material may increase old-oriented responses without necessarily increasing memory sensitivity.

The nonlinear arousal association should be interpreted descriptively rather than as confirmation of a specific theoretical law. Arousal-biased competition accounts and broader arousal–performance theories justify testing whether arousal-related associations vary across the arousal range, but they do not uniquely predict the upward curvature estimated here (Yerkes & Dodson, 1908; Aston-Jones & Cohen, 2005; Mather & Sutherland, 2011). The observed function should not be described as a classic inverted-U pattern or as evidence for an optimal arousal point. It indicates only that a constant linear slope was insufficient and that the association became more positive toward the higher end of participant-relative arousal. Because the specific shape and turning point were not specified in advance, the curve is best treated as a dataset-specific, exploratory pattern. Moreover, the present design cannot determine whether this pattern originated during encoding, consolidation, retrieval, or post-recognition appraisal. Independent replication with affective ratings obtained separately from the recognition test is needed before stronger psychological significance is attributed to the precise form of the curve.

The contrast between individualized valence and normative valence categories also requires careful interpretation. Linear individualized valence did not provide sufficient incremental explanatory value in the trial-level model, whereas valence-category differences were apparent in false-alarm rates, criterion c, and *d*′. These analyses address different questions. The continuous individualized predictor tests whether responses vary monotonically as a word is rated as more pleasant or more unpleasant relative to a participant’s own mean. In contrast, the categorical analyses compare negative, neutral, and positive word groups and can capture nonmonotonic contrasts, such as emotional-versus-neutral differences, that are not well represented by a single linear valence term. Prior work has reported U-shaped or category-dependent valence patterns (Gao et al., 2024), but the present response-surface model did not provide sufficient evidence to establish such a function at the trial level. The most defensible interpretation is therefore that valence was expressed differently across measurement levels and recognition outcomes, rather than that valence was behaviorally irrelevant.

Similarly, the absence of a conventional linear Valence × Arousal interaction should not be interpreted as evidence that valence and arousal are generally independent. Their joint contribution may depend on stimulus modality, task demands, memory measure, and functional form. Neural and behavioral studies have reported both partial separability and context-dependent interaction between these dimensions (Kensinger & Corkin, 2004; Mickley Steinmetz et al., 2010; Amini et al., 2026; Du Bruyn et al., 2026). The exploratory Valence × Arousal^2^ result raises the possibility that valence may relate to the curvature of the relationship between arousal and old-response confidence of the arousal association, rather than to its linear slope. However, this higher-order term was not part of the primary hypothesis, and the model-selection criteria did not agree. It should therefore be treated as a provisional lead rather than as a mechanistic finding. A stronger test would require preregistered functional forms, independently obtained ratings, and a sample designed to estimate nonlinear interactions.

The learning-task findings are most naturally interpreted within the levels-of-processing framework. Classifying a word as abstract or concrete requires semantic processing and may create a richer, more distinctive representation than the brief old/new recognition judgment used here (Craik & Lockhart, 1972; Craik & Tulving, 1975). Although retrieval practice can benefit later retention, its effectiveness depends on the operations engaged during retrieval (Rowland, 2014; Karpicke, 2017); in the present task, recognition judgments may have relied more on familiarity and less on semantic elaboration than abstract/concrete judgments. The semantic-judgment advantage therefore indicates that semantic elaboration was more effective than this particular recognition-judgment procedure under the present conditions, rather than that recognition practice is generally ineffective. This interpretation is consistent with evidence that learning effects vary with retention interval, task context, and available cognitive resources (Jia et al., 2024; Zheng et al., 2023), but it should remain anchored in the old-item analyses. Learning condition represented a genuine experimental history only for studied words, whereas the learning-condition labels assigned to new final-test items were analytic pseudo-labels used only for balancing. Accordingly, the learning-condition coefficient in the all-trial additive mixed-effects model should be treated as a design-control term rather than as a standalone estimate of the learning effect. The substantive evidence for the semantic-processing advantage comes from old-item confidence and hit-rate analyses. Similarly, the absence of a Learning Condition × Valence Category interaction indicates only that the present data did not detect clear valence-specific moderation, not that the learning benefit was necessarily identical across emotional categories.

The leave-one-participant-out analysis placed the inferential findings in a useful predictive context. Mixed-effects models test associations in the observed dataset while accounting for repeated measurements, whereas cross-validation asks whether the estimated fixed-effect structure retains information for a participant not used to fit the model. The above-chance performance indicates that some population-level ordering of responses generalized across participants. However, the very small difference between the retained arousal-based full model and the reduced comparison models shows that individualized arousal contributed little additional held-out information beyond item status and the basic fitted task structure. Statistical evidence for an arousal association should therefore not be equated with strong person-level prediction. The cross-validation analysis is best viewed as a robustness check that qualifies the practical magnitude of the effect rather than as a separate demonstration of predictive utility.

Several limitations define the boundaries of these interpretations. Most importantly, because affective ratings were collected after recognition, they cannot establish the temporal or causal role of arousal and valence; future work should obtain affective ratings independently of memory judgments, such as before encoding, in a separate session, or with counterbalanced rating and recognition orders. The nonlinear analysis and higher-order interactions were exploratory and not preregistered, and no a priori power analysis was conducted. Although the dataset included many trials, the number of participant clusters was modest for estimating small interactions and complex random-effects structures. In addition, the random-intercept-only models did not estimate participant- or item-specific variation in affective slopes, so the fixed effects should be interpreted as average associations under this restricted structure. Other limitations concern stimulus control, model specification, and generalizability. Normative familiarity differed modestly across categories, and lexical characteristics such as frequency, concreteness, and stroke count were unavailable, which may be relevant because the semantic task required abstract/concrete judgments. Sleep during the 24 h retention interval was not measured, despite its possible role in emotional-memory consolidation (Denis et al., 2022; Rawson & Jackson, 2024). Treating the six-point response scale as continuous did not directly model its ordinal structure, and the categorical signal-detection summaries did not provide a trial-level decomposition of confidence, sensitivity, and bias. Future research could use cumulative-link mixed models, hierarchical signal-detection models, ROC approaches, or diffusion models, and should estimate learning effects with an old-item-specific contrast or a Learning Condition × Item Status term so that genuine learning history is separated from pseudo-labels assigned to new items. Finally, findings from Chinese emotional words may not generalize directly to other stimulus types or memory systems.

Methodologically, the present study highlights the value of combining normative categories, individualized ratings, and complementary recognition outcomes. Normative ratings support stimulus selection and comparison with the broader literature, whereas participant-specific ratings capture within-category variation and allow trial-level associations to be estimated relative to each participant’s own affective scale. Similarly, confidence scores characterize the strength and direction of explicit recognition judgments, whereas false-alarm rates, criterion c, and *d*′ help distinguish response tendency from discrimination. No single measure fully identifies the underlying process, but their combination constrains interpretation more effectively than confidence alone. Within these boundaries, the study supports a differentiated rather than a unitary account of emotional recognition: participant-relative arousal was aligned with the subjective strength of old-oriented responding, valence-category differences were expressed more clearly in response bias and discrimination, and semantic elaboration was associated with stronger later recognition of studied words. The broader implication is not that one affective dimension universally dominates the other, but that conclusions about emotional memory depend on how emotion is measured, which recognition outcome is analyzed, and whether confidence, response tendency, and memory sensitivity are kept conceptually distinct.

## 5. Conclusions

The present study shows that emotional influences on recognition cannot be captured by a single measure. Participant-relative post-test arousal was more consistently associated with trial-level old-oriented response strength than individualized valence, particularly at higher arousal levels. In contrast, normative valence-category differences were more evident in false-alarm rates, response criterion, and *d*′. Together, these findings suggest that arousal and valence were expressed in different components of recognition: arousal was more closely related to the subjective strength of “old” responses, whereas valence was more apparent in response bias and discrimination.

Old-item analyses further showed that semantic-judgment learning was followed by higher old-item confidence and hit rates than recognition-judgment learning. This finding suggests that learning activities requiring semantic classification and elaboration may support delayed recognition of studied words more effectively than relatively simple recognition judgments. This implication is specific to the present task and should not be taken as evidence that retrieval practice is generally ineffective. Because learning-condition labels for new items were used only for analytic balancing, the interpretation of this learning advantage rests on the studied-item analyses.

These findings also have practical implications for evaluating memory reports. A confident “old” response may indicate correct recognition, but it may also be a confident false alarm. In educational assessment, eyewitness interviewing, clinical history taking, and health communication, confidence should therefore be considered alongside objective performance, corroborating evidence, or verification procedures. The participant-specific analyses also indicate that normative emotional categories may not fully capture how individuals experience the same material. Pretesting emotionally salient content with its intended audience may improve the design and interpretation of educational, clinical, and public-health communications.

These implications should remain proportional to the design. Because affective ratings were collected after recognition, they may have been influenced by familiarity, processing fluency, or confidence. The study therefore identifies associations and practical hypotheses rather than causal effects or validated interventions. Future research should obtain affective ratings independently of memory judgments and test these applications directly.

## Figures and Tables

**Table 1:** Participant-level descriptive statistics by learning condition and valence category.

Learning Condition	Valence Category	Old-Response Confidence	Hit Rate	False-Alarm Rate	d′	Criterion c
Semantic judgment	Negative	4.67 (0.63)	0.772 (0.141)	0.558 (0.194)	0.66 (0.43)	−0.49 (0.47)
Semantic judgment	Neutral	4.60 (0.63)	0.753 (0.141)	0.454 (0.182)	0.86 (0.47)	−0.30 (0.41)
Semantic judgment	Positive	4.73 (0.63)	0.776 (0.134)	0.508 (0.222)	0.77 (0.59)	−0.42 (0.48)
Recognition judgment	Negative	4.51 (0.69)	0.731 (0.152)	0.530 (0.216)	0.57 (0.61)	−0.39 (0.47)
Recognition judgment	Neutral	4.47 (0.77)	0.724 (0.180)	0.452 (0.178)	0.81 (0.52)	−0.28 (0.48)
Recognition judgment	Positive	4.58 (0.62)	0.750 (0.133)	0.501 (0.216)	0.72 (0.65)	−0.37 (0.42)

Note. Values are means with standard deviations in parentheses. *d*′ and criterion c were computed after applying the loglinear correction to hit and false-alarm proportions. Learning-condition labels for false-alarm rates, *d*′, and criterion *c* involving new items are analytic labels used only for balanced comparison.

**Table 2:** Non-intercept fixed-effect estimates for the retained mixed-effects model.

Parameter	β	SE	Wald χ^2^(1)	*p*	95% Wald CI
Arousal	0.0603	0.0142	17.95	<0.001	[0.032, 0.088]
Arousal^2^	0.0275	0.0101	7.46	0.006	[0.008, 0.047]
Learning: Semantic vs. Recognition	0.1209	0.0248	23.84	<0.001	[0.072, 0.169]
Status: New vs. Old	−1.0720	0.0630	289.38	<0.001	[−1.195, −0.948]

Note. Arousal was standardized within each participant, and Arousal^2^ was calculated from the standardized arousal scores. The model was fitted using restricted maximum likelihood and included crossed random intercepts for participant and stimulus item. β values are fixed-effect estimates, Wald tests use one degree of freedom, and confidence intervals are 95% Wald intervals. Learning condition was coded with recognition judgment as the reference level; item status was coded with old items as the reference level. The intercept is omitted because the table focuses on the substantive predictors.

## Data Availability

The data that support the findings of this study are available from the corresponding author upon reasonable request. The data are not publicly available because they contain participant-level behavioral responses and individualized affective ratings.

## Supplementary Materials

The following supporting information can be downloaded at: https://www.mdpi.com/article/10.3390/bs16091594/s1, Supplementary Analysis S1: Candidate Linear Mixed-Effects Models.
