# Supplementary material for: The Effects of Individualized Arousal on Recognition Memory of Words
Source: Behav Sci (Basel). 2026 Sep 7;16(9):1594. doi: 10.3390/bs16091594 (PMC13603306; doi:10.3390/bs16091594)
Supplement: Supplementary file 1 [file behavsci-16-01594-s001.zip › behavsci-4443542-supplementary.pdf]

# Supplementary Materials

## The Effects of Individualized Arousal on Recognition Memory of Words

**Wenling Zhang [1] and Xi Jia [1,*]**

## Supplementary Analysis S1. Candidate Linear Mixed-Effects Models

This supplementary analysis reports the complete model definitions, maximum-likelihood comparisons, and restricted-maximum-likelihood fixed-effect estimates for the five candidate linear mixed-effects models. The analysis used 14,897 valid trials from 40 participants and 378 final-test stimulus items.

Individualized arousal and valence ratings were standardized separately within each participant, and quadratic terms were calculated from the standardized predictors. Recognition judgment was the reference level for learning condition, and old items were the reference level for item status. All models included crossed random intercepts for participant and stimulus item. Models with different fixed-effect structures were fitted by maximum likelihood for comparison; the same models were then fitted by restricted maximum likelihood for coefficient estimation.

The learning-condition labels assigned to new items were used only for analytic balancing and did not represent actual learning histories. Accordingly, the learning-condition coefficient in the all-trial models is treated as a design-control term rather than as an independent estimate of an encoding effect on new items.

## Candidate model formulas

**M1 (Arousal-based):** Response ~ Arousal_z + $\text{Arousal\_z}^2$ + Learning + Status + (1 | Participant) + (1 | Item)

**M2 (Additive valence):** Response ~ Arousal_z + $\text{Arousal\_z}^2$ + Valence_z + Learning + Status + (1 | Participant) + (1 | Item)

**M3 (Linear interaction):** Response ~ Arousal_z + $\text{Arousal\_z}^2$ + Valence_z + Valence_z × Arousal_z + Learning + Status + (1 | Participant) + (1 | Item)

**M4 (Quadratic response surface):** Response ~ Arousal_z + $\text{Arousal\_z}^2$ + Valence_z + $\text{Valence\_z}^2$ + Valence_z × Arousal_z + Learning + Status + (1 | Participant) + (1 | Item)

**M5 (Exploratory nonlinear interaction):** Response ~ Arousal_z + $\text{Arousal\_z}^2$ + Valence_z + Valence_z × Arousal_z + Valence_z × $\text{Arousal\_z}^2$ + Learning + Status + (1 | Participant) + (1 | Item)

## Maximum-likelihood model comparisons

The comparison results supported the arousal-based model as the primary reporting model. Adding linear valence to M1 did not improve fit, and adding the conventional Valence × Arousal interaction to M2 also did not improve fit. The quadratic response-surface model did not improve fit relative to M1. M5 improved the likelihood-ratio comparison and reduced AIC relative to M3, but BIC increased; because this higher-order interaction was exploratory and the information criteria disagreed, M1 was retained as the primary model.

**Table S1.** Maximum-likelihood fit indices and nested comparisons for the candidate models.

**Panel A.** Model fit indices

| Model | Description | Fixed-effect structure | k | logLik | AIC | BIC |
|---|---|---|---|---|---|---|
| M1 | Arousal-based | Arousal + Arousal² + Learning + Status | 8 | -27613.065 | 55242.130 | 55303.001 |
| M2 | Additive valence | M1 + Valence | 9 | -27612.662 | 55243.324 | 55311.804 |
| M3 | Linear interaction | M2 + Valence × Arousal | 10 | -27612.550 | 55245.100 | 55321.189 |
| M4 | Quadratic response surface | Arousal + Arousal² + Valence + Valence² + Valence × Arousal + Learning + Status | 11 | -27612.343 | 55246.686 | 55330.384 |
| M5 | Exploratory nonlinear interaction | M3 + Valence × Arousal² | 11 | -27609.179 | 55240.358 | 55324.056 |

**Panel B.** Nested likelihood-ratio comparisons

| Comparison | Added term(s) | Δdf | LRT $\chi^2$ | p | ΔAIC | ΔBIC |
|---|---|---|---|---|---|---|
| M2 vs. M1 | Valence | 1 | 0.81 | .369 | +1.19 | +8.80 |
| M3 vs. M2 | Valence × Arousal | 1 | 0.22 | .636 | +1.78 | +9.39 |
| M4 vs. M1 | Valence, Valence², and Valence × Arousal | 3 | 1.44 | .695 | +4.56 | +27.38 |
| M5 vs. M3 | Valence × Arousal² | 1 | 6.74 | .009 | -4.74 | +2.87 |

Note. k is the number of estimated parameters, including fixed-effect coefficients, the participant and item random-intercept variances, and the residual variance. Positive ΔAIC or ΔBIC values indicate worse fit for the larger model; negative values indicate improvement.

## Restricted-maximum-likelihood fixed-effect estimates

Table S2 reports the fixed-effect coefficients from the REML fits. Each Wald test evaluates one coefficient with one degree of freedom, and the confidence intervals are 95% Wald intervals. The higher-order Valence × Arousal² term in M5 is exploratory.

**Table S2.** REML fixed-effect estimates for all candidate linear mixed-effects models.

| Model | Description | Parameter | Estimate β | SE | Wald $\chi^2(1)$ | p | 95% CI |
|---|---|---|---|---|---|---|---|
| M1 | Arousal-based | Intercept | 4.5073 | 0.0981 | 2112.47 | < .001 | [4.315, 4.699] |
| M1 | Arousal-based | Arousal | 0.0603 | 0.0142 | 17.95 | < .001 | [0.032, 0.088] |
| M1 | Arousal-based | Arousal² | 0.0275 | 0.0101 | 7.46 | .006 | [0.008, 0.047] |
| M1 | Arousal-based | Learning-condition label: Semantic vs. Recognition | 0.1209 | 0.0248 | 23.84 | < .001 | [0.072, 0.169] |
| M1 | Arousal-based | Item status: New vs. Old | -1.0720 | 0.0630 | 289.38 | < .001 | [-1.195, -0.948] |
| M2 | Additive valence | Intercept | 4.5068 | 0.0980 | 2114.89 | < .001 | [4.315, 4.699] |
| M2 | Additive valence | Arousal | 0.0584 | 0.0144 | 16.47 | < .001 | [0.030, 0.087] |
| M2 | Additive valence | Arousal² | 0.0277 | 0.0101 | 7.54 | .006 | [0.008, 0.047] |
| M2 | Additive valence | Valence | 0.0168 | 0.0187 | 0.81 | .369 | [-0.020, 0.053] |
| M2 | Additive valence | Learning-condition label: Semantic vs. Recognition | 0.1209 | 0.0248 | 23.82 | < .001 | [0.072, 0.169] |
| M2 | Additive valence | Item status: New vs. Old | -1.0712 | 0.0631 | 288.35 | < .001 | [-1.195, -0.948] |
| M3 | Linear interaction | Intercept | 4.5071 | 0.0983 | 2103.58 | < .001 | [4.314, 4.700] |
| M3 | Linear interaction | Arousal | 0.0593 | 0.0145 | 16.69 | < .001 | [0.031, 0.088] |
| M3 | Linear interaction | Arousal² | 0.0266 | 0.0103 | 6.60 | .010 | [0.006, 0.047] |
| M3 | Linear interaction | Valence | 0.0137 | 0.0198 | 0.48 | .491 | [-0.025, 0.053] |
| M3 | Linear interaction | Valence × Arousal | 0.0062 | 0.0132 | 0.22 | .636 | [-0.020, 0.032] |
| M3 | Linear interaction | Learning- | 0.1209 | 0.0248 | 23.84 | < .001 | [0.072, 0.169] |

| | | | | | | | |
|---|---|---|---|---|---|---|---|
| | | condition label: Semantic vs. Recognition | | | | | |
| M3 | Linear interaction | Item status: New vs. Old | -1.0706 | 0.0631 | 288.00 | < .001 | [-1.194, -0.947] |
| M4 | Quadratic response surface | Intercept | 4.5008 | 0.0986 | 2081.56 | < .001 | [4.307, 4.694] |
| M4 | Quadratic response surface | Arousal | 0.0559 | 0.0155 | 13.03 | < .001 | [0.026, 0.086] |
| M4 | Quadratic response surface | $Arousal^2$ | 0.0247 | 0.0107 | 5.30 | .021 | [0.004, 0.046] |
| M4 | Quadratic response surface | Valence | 0.0148 | 0.0199 | 0.55 | .457 | [-0.024, 0.054] |
| M4 | Quadratic response surface | $Valence^2$ | 0.0084 | 0.0131 | 0.41 | .521 | [-0.017, 0.034] |
| M4 | Quadratic response surface | Valence × Arousal | 0.0047 | 0.0134 | 0.13 | .722 | [-0.021, 0.031] |
| M4 | Quadratic response surface | Learning-condition label: Semantic vs. Recognition | 0.1210 | 0.0248 | 23.88 | < .001 | [0.072, 0.170] |
| M4 | Quadratic response surface | Item status: New vs. Old | -1.0711 | 0.0631 | 288.44 | < .001 | [-1.195, -0.947] |
| M5 | Exploratory nonlinear interaction | Intercept | 4.5043 | 0.0980 | 2111.63 | < .001 | [4.312, 4.696] |
| M5 | Exploratory nonlinear interaction | Arousal | 0.0526 | 0.0147 | 12.73 | < .001 | [0.024, 0.082] |
| M5 | Exploratory nonlinear interaction | $Arousal^2$ | 0.0319 | 0.0105 | 9.15 | .002 | [0.011, 0.053] |
| M5 | Exploratory nonlinear interaction | Valence | -0.0126 | 0.0223 | 0.32 | .572 | [-0.056, 0.031] |
| M5 | Exploratory nonlinear interaction | Valence × Arousal | -0.0036 | 0.0137 | 0.07 | .793 | [-0.030, 0.023] |
| M5 | Exploratory nonlinear interaction | Valence × $Arousal^2$ | 0.0214 | 0.0083 | 6.74 | .009 | [0.005, 0.038] |
| M5 | Exploratory nonlinear interaction | Learning-condition label: Semantic vs. Recognition | 0.1212 | 0.0248 | 23.96 | < .001 | [0.073, 0.170] |

| | | | | | | | |
|---|---|---|---|---|---|---|---|
| M5 | Exploratory nonlinear interaction | Item status: New vs. Old | -1.0699 | 0.0630 | 288.18 | < .001 | [-1.193, -0.946] |

Note. Arousal and valence were standardized within participant. "Learning-condition label" contrasts semantic with recognition judgment, and "Item status" contrasts new with old items. Because new-item condition labels were analytic pseudo-labels, the learning coefficient is interpreted as a nuisance design-control term in the all-trial models. All models included crossed random intercepts for participant and stimulus item.

## Interpretation

The candidate-model results support the arousal-based model as the primary summary of the trial-level old-oriented response scores. Linear individualized valence and the conventional Valence × Arousal interaction did not add reliable explanatory value beyond arousal, learning-condition label, and item status. The exploratory Valence × $Arousal^2$ term was significant in M5 and improved AIC relative to M3, but BIC favored the more parsimonious model. This higher-order interaction should therefore be treated as a supplementary finding requiring preregistered replication.
